# Primary HIV-1 infection presenting with nephrotic-range proteinuria and severe acute kidney injury mimicking imported Lassa fever

**DOI:** 10.1007/s15010-024-02466-9

**Published:** 2025-02-18

**Authors:** Frieder Pfäfflin, Ralf Schindler, Miriam Songa Stegemann, Wolfgang Schneider, Leif Erik Sander, Philipp Enghard, Stephan Achterberg, Dirk Schürmann

**Affiliations:** 1 Department of Infectious Diseases and Critical Care Medicine, Charité - Universitätsmedizin Berlin, Corporate member of Freie Universität Berlin and Humboldt-Universität zu Berlin, Charitéplatz 1, 10117 Berlin, Germany; 2 Nierenzentrum Luckenwalde, Weststrasse 16, 14943 Luckenwalde, Germany; 3 Department of Pathology, Charité - Universitätsmedizin Berlin, Corporate Member of Freie Universität Berlin and Humboldt-Universität zu Berlin, Berlin, Germany; 4 Department of Nephrology and Medical Intensive Care, Charité - Universitätsmedizin Berlin, Corporate Member of Freie Universität Berlin and Humboldt-Universität zu Berlin, Berlin, Germany

**Keywords:** Primary HIV infection, Nephrotic-range proteinuria, Minimal change disease, Acute kidney injury, Hemodialysis

## Abstract

**Purpose:**

Primary HIV-1 infection (PHI) can present with protean clinical manifestations. We report a rare presentation of PHI that underscores that a high index of suspicion is required for diagnosis of PHI.

**Methods:**

We report on a 54-yearold previously healthy woman of African descent who presented with sudden-onset nephrotic-range proteinuria and acute kidney injury (AKI) requiring hemodialysis in the setting of febrile multiple organ dysfunction syndrome. Both the epidemiological and clinical features initially pointed to imported Lassa fever, but this was ruled out. She was eventually diagnosed with PHI. We reviewed the literature for other patients who presented with PHI and AKI requiring hemodialysis.

**Results:**

Kidney biopsy evaluation, including conventional and electron microscopy, revealed minimal change disease (MCD) and diffuse tubular damage leading to AKI. To date, MCD has not been reported to be associated with PHI and severe AKI. A literature search revealed six additional cases of severe PHI-associated AKI requiring hemodialysis. In four cases, severe rhabdomyolysis with tubulotoxic myoglobinuria played the primary causative role, while in one case each AKI was associated with HIV-associated nephropathy (HIVAN) and hemolytic uremic syndrome, respectively.

**Conclusions:**

Severe AKI requiring hemodialysis is a rare manifestation of PHI and may be associated with several conditions, most commonly PHI-associated rhabdomyolysis with tubulotoxic myoglobinuria. Severe AKI in PHI may also occur as a complication of MCD manifesting with nephrotic-range proteinuria. PHI should be considered in the differential diagnosis in patients presenting with severe proteinuria and AKI in the setting of febrile multiple organ dysfunction syndromes, including hemorrhagic fever diseases.

## Introduction

Acute retroviral syndrome during primary HIV infection (PHI) usually manifests as an unspecific flu-like or mononucleosis-like illness [1, 2]. The immune-mediated inflammatory acute retroviral syndrome is caused by a fierce cytokine response (cytokine storm) and presents with unspecific symptoms such as fever, weakness, myalgia, headache, cough, pharyngitis, and rash [1–3]. Organ involvement is considered to result from toxic immune reactions or toxic virus effects, secondary opportunistic infections or complications from concurrent diseases [2]. Severe acute kidney injury (AKI) is an uncommon complication of PHI [4].

We report a patient with PHI who presented with nephrotic-range proteinuria and AKI requiring hemodialysis. We also reviewed the literature and data from other patients with PHI who presented with AKI and required hemodialysis.

## Case report

A 54-yearold previously healthy woman of Ghanaian descent returned from a 4-week Ghana stay visiting friends and relatives in a rural area in Ashanti District. Three weeks after her return, she presented with an 8-day history of sore throat, cough, fever, loss of appetite, nausea and vomiting, general progressive weakness and fatigue, headache, severe back, muscle and limb pain, watery diarrhea, and dark coloured urine without dysuria. Four days before admission, treatment with ibuprofen for pain relief and with ampicillin for suspected respiratory infection had been started. An HIV antibody test had been negative two years earlier. The patient´s medical history to date had been unremarkable. The patient denied any sexual contact or any other risk for HIV infection in the last two years.

The preliminary diagnosis was Lassa fever, a viral hemorrhagic fever (VHF) disease. The International Society for Infectious Diseases had announced an investigation into a Lassa fever outbreak in the Ashanti region during the patient´s stay [5]. Signs and symptoms, laboratory findings, region of potential exposure and incubation period were compatible with Lassa fever [6].

Physical examination revealed fever (39.0 °C), tachycardia (116 beats/minute), blood pressure of 100/60 mmHg, and ulcerous pharyngitis, inconspicuous lymphnode status, no orbital or leg oedema, no kidney palpitations. Chest x-ray was unremarkable. Heart, lung and abdominal ultrasound examination revealed normal cardial ejection fraction, no pericardial or pleural effusion, no hepatosplenomegaly, symmetrically enlarged/swollen kidneys with very echo-rich parenchyma consistent with AKI, normal perfusion.

Pertinent blood laboratory parameters were: erythrocytes and hemoglobin normal, hematocrit 0.39 L/L (normal 0.35–0.45), leucocytes 3490/µL (normal 4500–11,000), lymphocytes 1010/µL (normal 1400–3700), thrombocytes normal, alanine aminotransferase 130 U/L (normal < 34), aspartate aminotransferase 276 U/L (normal < 35), creatinine 6.8 mg/dL (normal < 1), urea 191 mg/dL (normal 14–46), lactate dehydrogenase 1752 U/L (normal < 247), bilirubin normal, haptoglobin normal, lipase 1398 U/L (normal < 70), skeletal muscle creatine phosphokinase (CPK) 912 U/L (normal < 145), myoglobin 784 µg/L (normal < 70), coagulation parameters normal, c-reactive protein 1.2 mg/dL (normal < 0.5), albumin 2.5 g/dL (normal 3.5–5.2), cholesterol 183 mg/dL (normal < 200), ferritin 10,800 µg/L (normal 30–400). Immune diagnostics negative, estimated glomerular filtration rate (eGFR): 5.7 ml/min indicating kidney failure (normal > 90 ml/min).

The urine dipstick test showed blood and leucocytes each 1+, protein 3+, and nitrite negative. Automated urine analysis revealed > 250 red blood cells and leucocytes per µL each, a small number of renal tubular cells, squamous epithelial cells, hyaline cylinders and bacteria. No qualified urine sediment microscopy was performed. Urine osmolarity was 317 mOsm/kg (normal 50–1400). Urine chemistry: proteinuria 20 g/L (normal < 0.12), with an albumin share of 19.3 g/L (normal < 0.02), urine protein to creatinine ratio (UPCR) 18.0 g/g (20 g/L protein to 1.06 g/L creatine) indicating nephrotic-range proteinuria (defined by a UPCR of > 3.5 g/g), normal UPCR < 0.15 g/g. Urine culture: at least three undifferentiated bacteria considered to be contaminants.

Blood cultures were negative. Stool cultures were positive for *Escherichia coli* and *Klebsiella pneumoniae* considered to be colonizers. Further work-up for infectious diseases remained negative: malaria (negative rapid diagnostic test, negative thick and thin blood films), dengue (negative rapid diagnostic test), leptospirosis and syphilis (negative serologic tests), Lassa fever, Ebola virus disease, Marburg virus disease, Crimean-Congo hemorrhagic fever, yellow fever, hepatitis viruses, herpes viruses (negative polymerase chain reaction test).

Finally, the HIV ELISA screening test was reactive. The CD4 count was 306/µL with a CD4/CD8 ratio of 1.03. The viral load of HIV was very high with > 10,000,000 HIV-1 RNA copies/mL plasma. PHI was diagnosed. The confirmative Western blot assay was initially still negative (all bands nonreactive) and became positive with reactive diagnostic bands four weeks later.

**Fig. 1:**
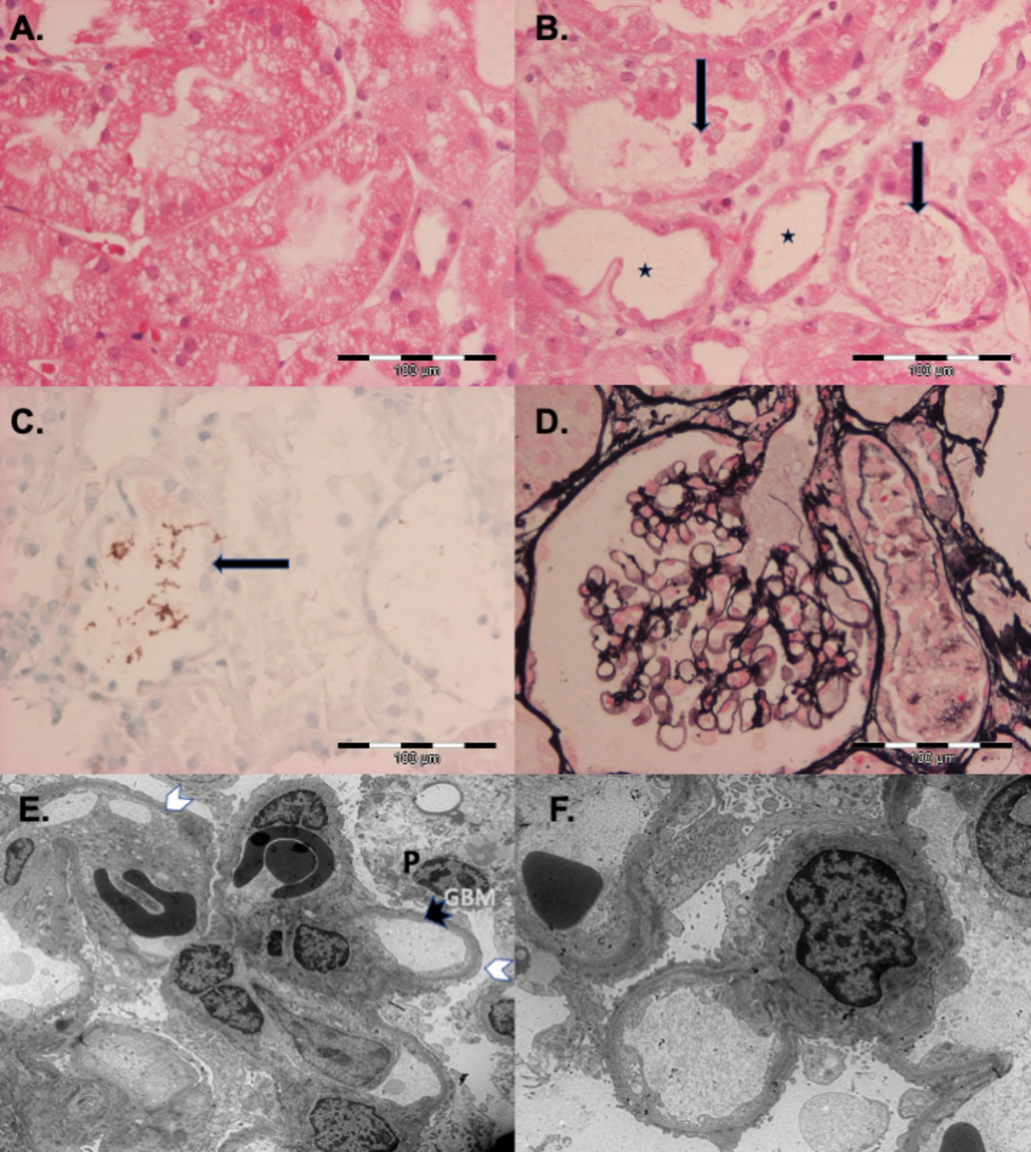
Kidney biopsy of the patient demonstrating acute tubular injury (ATI) and glomerular minimal change glomerulopathy (MCD). **A** Diffuse coarse tubular vacuoles (ATI) (hematoxylin–eosin stain). **B** Focal ATI with dilated tubules and attenuation of proximal tubular cells (asterisks) and denuded cells or debris in tubular lumen (arrows) (hematoxylin–eosin stain). **C** Myoglobin in the tubular lumen (arrow). **D** Jones silver stain demonstrating normal glomeruli. **E** Electron microscopy showing diffuse foot process effacement (white arrowheads) of podocytes (P), unremarkable glomerular basement membranes (GBM) (black arrowhead), without signs of deposits. **F** Electron microscopy showing diffuse foot process effacement and microvillous transformation

A kidney biopsy was performed at the day of presentation (Fig. 1). Histological and immunohistological examinations revealed non-inflammatory diffuse tubular epithelial damage with segmental epithelial necrosis, scattered tubular myoglobin cylinders, and intact normocellular glomeruli with intact vascular loops without immune complex deposits. Electron microscopy revealed glomerular diffuse effacement of visceral podocyte foot processes, microvillous transformation consistent with a minimal change disease (MCD). There were no tubuloreticular inclusions or mesangial lesions.

**Fig. 2:**
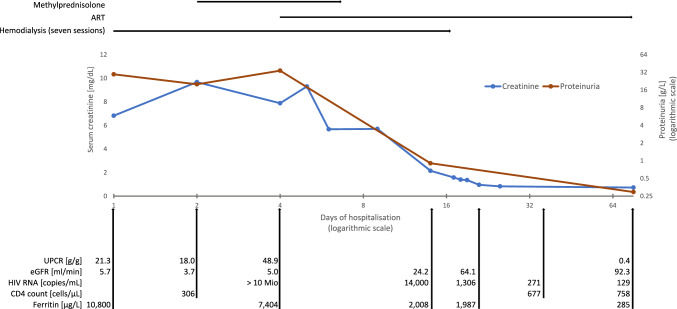
Therapeutic interventions and key laboratory parameters during the hospital stay of a patient with PHI presenting with nephrotic-range proteinuria and severe AKI. Seven hemodialysis sessions were carried out from day 1 to day 16. Combination ART (boosted darunavir plus abacavir and lamivudine) was started on day 4. Methylprednisolone (0.5 g per day) was started on day 2 and administered for seven days because of extreme hyperferritinemia with suspected HLH, which was, however, finally not confirmed. For normal values of laboratory parameters, see case report. For further explanations, see article. *ART* antiretroviral therapy, *eGFR* estimated glomerular filtration rate, *HLH* hemophagocytic lymphohistiocytosis, *PHI* primary HIV infection, *UPCR* urine protein creatinine ratio

Because of critical azotaemia with an increase of serum creatinine from 6.8 mg/dL at presentation to 9.7 mg/dL (eGFR: 3.7 ml/min) on hospital day 2, hemodialysis using a jugular catheter was initiated. The administered treatment and the course of pertinent laboratory values is shown in Fig. 2. After seven hemodialysis sessions renal function recovered completely within four weeks except for mild proteinuria that lasted over eleven weeks until discharge with loss to follow-up. Urine protein was 0.29 g/L (normal < 0.12), with an albumin share of 0.17 g/L (normal < 0.02). The UPCR was 0.39 g/g (normal < 0.15 g/g). Antibiotic treatment with ceftriaxone was initially started for suspected urinary tract infection for three days.

Standard antiretroviral therapy adapted for renal insufficiency/hemodialysis with norvir-boosted darunavir plus abacavir/lamivudine was initiated (after ruling out HLA B57) from day 4 of hospitalization, resulting in a rapid decline of the plasma HIV RNA (Fig. 2). Initially, high-dose steroids were also administered for seven days because of the presence of hyperferritinemia, which was considered possibly indicative of life-threatening hemophagocytic lymphohistiocytosis (HLH) [7]. The course was temporarily complicated by the occurrence of self-limiting mild hemolytic anemia (no blood transfusions needed) and a suspected bacterial infection without pathogen detection with consecutive antibiotic therapy.

## Discussion

We report a previously healthy female patient with PHI who presented with sudden development of nephrotic-range proteinuria and AKI requiring hemodialysis. PHI was diagnosed by a reactive screening ELISA serum antibody test with very high plasma HIV-1 RNA and a negative Western blot test, that later turned positive. Severe proteinuria and severe AKI are rare manifestations of PHI [2, 4]. The diagnosis of PHI presenting with such unusual manifestations requires a high index of suspicion.

Nephrotic-range proteinuria was detected with a diagnostic UPCR of 18.0 g/g on admission, with a worsening UPCR of 47.6 g/g and an albuminuria of 36.2 g/L on day 4 leading to a drop of the serum albumin from 2.5 at admission to 1.8 g/dL on day 4 (normal 3.5–5.2). It is noteworthy that the patient did not yet show the typical leg and eyelid edema associated with nephrotic-range proteinuria, which, together with the still normal serum cholesterol, indicates a sudden development of proteinuria.

Because of unexplained severe proteinuria and AKI a kidney biopsy was performed. Electron microscopy revealed MCD, which typically remains undetected by light microscopy, as the morphologic correlate of nephrotic-range proteinuria [8, 9]. MCD is an established cause of nephrotic-range proteinuria and complicating AKI up to the need of hemodialysis [8, 9]. Light microscopy revealed diffuse tubular damage with segmental necrosis as histological correlate of AKI with azotemia. MCD with proteinuria and AKI has been infrequently reported in patients with chronic HIV infection, but has — to the best of our knowledge – not yet been report in patients with PHI.

In a series of eight HIV-infected patients with MCD there was one in whom MCD was diagnosed at the time of HIV diagnosis, but it remains unclear whether MCD was diagnosed with chronic or acute PHI [10]. The patient had an HIV viral load of 47,500/mL, which is in the range typically seen in patients with chronic HIV infection. However, the high CD4 cell count (450/µL) may indicate that the primary infection was recent. MCD has been reported in seven other patients with obviously chronic HIV infection, all on ART [10]. This suggests that, albeit rare, MCD can occur in all stages of HIV infection, from PHI with high viral replication to chronic HIV infection with or without ART and suppressed viral replication. It is noteworthy that only three of these patients presented with AKI, which was mild in two cases and moderate in one [10].

We believe that MCD and complicating AKI in our patient was multifactorial in origin. MCD can be associated with viral infections [8, 9] and also with drugs such as ibuprofen and ampicillin, which the patient had both taken shortly prior to presentation. AKI with tubular damage is a typical complication of MCD with nephrotic-range proteinuria [8, 9], but there were also other nephrotoxic factors present known to cause AKI, including hypovolemia due to vomiting, diarrhea and fever, and short-term drug use (ibuprofen, ampicillin).

Organ involvement during PHI in our patient included rhabdomyolysis. Rhabdomyolysis is a rare manifestation of PHI and severe myoglobinuria has been reported to lead to AKI and even the need for renal replacement therapy [11, 12] (Table 1, patients 2, 4–6). Severe tubulotoxic myoglobinuria is an established cause of AKI manifesting with severe tubulopathies [13]). However, rhabdomyolysis in our patient was rather mild at presentation (CPK 912 U/L) and myoglobinuria most probably played only a minor contributory role to AKI, even if scattered tubular myoglobin cylinders were detected in the renal biopsy taken on the day of admission (Fig. 1). No myoglobin was detected in the urine, which was analyzed for myoglobin for the first time three days after admission. AKI due to rhabdomyolysis requiring hemodialysis is usually observed only in severe rhabdomyolysis. In a cohort of patients without HIV infection, CPK was usually > 5000 U/L [13].

**Table 1:** Patients with Primary HIV Infection (PHI) presenting with severe acute renal kidney injury (AKI) requiring hemodialysis

Case number, first author, year (reference)	Age (years); race; gender	CD4 cell count/µL; HIV RNA/mL plasma	Azotemia: s-creatinine (mg/dL); s-urea or s-urea nitrogen (BUN) (mg/dL)	Data from urinalysis	Concurrent conditions		Kidney biopsy	Renal Outcome	Patient outcome, antiretroviral therapy (ART) initiated
					PHI-associated rhabdomyolysis (yes/no); S-CPK (U/L)	Others			
1, Szabo S, 2002 [14]	47, African female	CD4 cells not reported; > 75,000	14; s-urea nitrogen 169	21.4 g protein in 24- hour urine collection	No	Cavitary lesion left lung, ethanol abuse, untreated hypertension	Collapsing focal segmental glomerulosclerosis consistent with HIVAN	No detailed information given	Response of lung lesion to antibiotic treatment, recovered, ART until 2 weeks after discharge, died 4 months later due to endocarditis and sepsis
2, Prahabar MR, 2008 [15]	42, Saudi male	436; 638,800	6.8	Dipstick: blood positive, protein 4 + , myoglobin in urine positive	Yes; 278,000	Occasional ethanol use	Congested glomeruli, interstitial infiltrates, acute tubular necrosis, intratubular casts	Recovery	Recovery within 1-month follow-up after discharge, no ART
3, Gomes AM, 2009 [16]	38, black male	308; > 1,000,000	7.5; s-urea 195	No data given	No	Hemolytic uremic syndrome	Not done	Recovery	Improvement until discharge, no ART
4, Merrill ER, 2011, [17]	19, African American male	290; 273,000	8.0; s-urea nitrogen 54	Dipstick: protein 3 + , blood 3 + , no casts, myoglobin in urine demonstrated	Yes; 234,417	Possibly active CMV infection (CMV IgG positive)	Normal glomeruli, acute tubular necrosis, myoglobin containing casts, diffuse dilatation of the proximal tubules, granular eosinophilic casts	Improvement but still requiring hemodialysis at discharge	Improvement, no follow-up after discharge, no ART
5, Moanna A, 2011, [18]	19, African American male	34; > 750,000	8.0	Urinalysis revealed protein und blood in urine	Yes; > 16,000	*Streptococcus mitis* bacteremia	Not done	Recovery within 6 weeks	Antibiotic treatment of bacteremia, recovered, no ART
6, Noe MM, 2017 [19]	24, African American male	170; > 10,000,000	5.7	Myoglobin in urine +	Yes: > 200,000	None reported	Not done	Improvement but still requiring hemodialysis at discharge	Improvement, no follow-up after discharge, ART
7, Pfäfflin F 2025, case presented in this article	54, Woman of African descent	306; > 10,000,000	6.8 with an increase to 9.7 on hospital day 2; s-urea 191	Dipstick: protein 3 + ; 20 g/L protein in urine with a share of 19.3 g/L albumin, further results see Case Report	Yes; 912	Ibuprofen use hypovolemia (vomiting, diarrhea)	Minimal change disease (MCD), non-inflammatory diffuse tubular damage with segmental epithelial necrosis, myoglobin casts in tubuli	Recovery with persistent mild proteinuria until discharge	Recovery within 11 weeks until discharge, during the inpatient course suspected sepsis and mild hemolysis, ART

All patients with a PHI diagnosis had initially either a negative HIV-ELISA test or, if positive, negative or still incomplete HIV-Western blot results, which became confirmative of HIV infection during the further course. Information and data in the table where reported

*PHI* Primary HIV Infection, *S-CPK* serum creatine phosphokinase (normal at Charité < 145 U/L), *s-creatinine* serum creatinine (normal at Charité < 1 mg/dL), *s-urea* serum urea (normal at Charité 14–46 mg/dL), *s-urea nitrogen (BUN)* 0.446 × s-urea, *HIVAN* HIV-associated nephropathy

Severe AKI in patients with PHI requiring renal replacement therapy has rarely been reported. We performed a literature search in the PubMed database using the terms PHI, AKI, hemodialysis, and renal replacement therapy. We also reviewed the cited literature with case reports and review articles on PHI and AKI. Another six patients were found by July 2024 (Table 1, cases 1–6). In four of six patients (67%) severe PHI-associated rhabdomyolysis with tubulotoxic myoglobinuria was the primary cause of AKI (Table 1, patients 2, 4, 5, and 6). Of the remaining two cases of PHI-associated AKI requiring hemodialysis, one was associated with hemolytic uremic syndrome and the other with HIV-associated nephropathy (HIVAN), which presented histologically as collapsing focal segmental glomerulosclerosis (Table 1, cases 1 and 3). HIVAN, which often leads to end-stage renal disease, is a common complication of HIV infection but usually occurs in the setting of chronic HIV infection [20].

Clinical differentiation of types and various pathological manifestations of glomerulopathies and tubulopathies in patients with PHI who present with symptoms such as proteinuria, hematuria, and AKI can be difficult and may require renal biopsy [4, 11] (Table 1, patients 1, 2, 4, and 7). Patients with severe tubulotoxic myoglobinuria caused by rhabdomyolysis, which appears to be the most common cause of severe proteinuria in PHI-associated AKI [12] (Table 1), can be readily distinguished by significant rhabdomyolysis parameters such as serum creatinine and myoglobin, and by urinalysis including identification of myoglobin as component of proteinuria. Myoglobin is filtered by the glomeruli when large amounts are excreted, exceeding the binding capacity of plasma protein, and can eventually lead to tubule obstruction and renal dysfunction. In our patient, MCD was ultimately diagnosed as the cause of the severe proteinuria only after a biopsy that included conventional histology and electron microscopy.

The PHI-associated retroviral syndrome, which is characterized by multiorgan involvement, is self-limiting unless it is complicated by factors that affect prognosis, such as severe secondary infection or concomitant illness. A number of factors may have contributed to the resolution of MCD with severe proteinuria and AKI in our patient after initial support with renal replacement therapy. The early initiation of antiretroviral therapy suppressed viral replication and rapidly decreased the viral load, attenuating the hyperinflammatory retroviral syndrome. In addition, the elimination of potentially nephrotoxic factors that may have contributed to the development of MCD and AKI, such as ibuprofen and ampicillin, may have contributed to the resolution. The short course of high-dose steroids at the onset of the illness may have also played a role in the resolution of MCD, as steroids are the primary treatment for MCD [8, 9]. High-dose steroid therapy was originally administered because extreme hyperferritinemia suggested the possibility of life-threatening HLH. Extreme hyperferritinemia and HLH are unsual manifestations of PHI [7, 21]. Ultimately, HLH was not confirmed because the required criteria for diagnosis were not met [22].

PHI may be a differential diagnosis of VHF [23]. At the time of presentation, our patient was thought to be suffering from imported Lassa fever and strict infection prevention and control measures were put in place. Lassa fever is a potentially fatal disease that is endemic in rural areas of Ghana. An outbreak of Lassa fever had been reported in the area where the patient had stayed before travelling to Germany [5]. Signs and symptoms of Lassa fever are unspecific and include fever, pharyngitis, and proteinuria. AKI requiring hemodialysis may also occur. Thus, both the epidemiological and clinical features in the present patient were compatible with Lassa fever [6]. However, in contrast to our patient, renal biopsies in Lassa fever patients have shown glomerular necrosis instead of MCD in addition to tubular necrosis [24].

In summary, PHI should be considered in patients with fever of unknown origin, proteinuria and AKI and in patients whose presentation and epidemiological history are compatible with Lassa fever. PHI should also be considered if a possible previous risk of HIV transmission is denied. A kidney biopsy, including an electron microscopic examination, may be needed to determine the complicating secondary renal disease.

## Data Availability

The patient file is not publicly accessible for legal reasons. All data obtained and analyzed are included in this article. Further inquiries can be directed to the corresponding author.
